# Cancer‐associated fibroblast heterogeneity in chordoma^†^

**DOI:** 10.1002/path.6420

**Published:** 2025-03-15

**Authors:** Jack C Henry, Angus J M Cameron

**Affiliations:** ^1^ Barts Cancer Institute, Queen Mary University of London, John Vane Science Centre, Charterhouse Square London UK

**Keywords:** cancer‐associated fibroblasts, chordoma, tumour invasion, single‐cell transcriptomics, spatial transcriptomics

## Abstract

In solid tumours, malignant cells develop relationships with nonmalignant stromal cells to support tumour growth, tissue structure, and nutrient supply. In a recent issue of this journal, Zheng and Guo catalogue the cellular landscape in chordoma using a combination of single‐cell and spatial transcriptomics. Despite the mesenchymal nature of chordoma, malignant cells retain expression of epithelial markers, in addition to mesenchymal, partial‐EMT, and stem‐cell signatures. Cancer‐associated fibroblasts (CAFs) are among the most abundant stromal cells and the authors define an inflammatory CAF subtype (iCAF), which is associated with poor outcome and tumour invasion. It is proposed that iCAFs arise from normal fibroblasts during malignant tumour progression and play a causative role in driving an invasive poor prognosis tumour phenotype. Recent reports by this and other groups have separately catalogued cell populations, including CAFs and immune cells in chordoma. The next challenge will be to integrate findings from these distinct studies to allow a consensus to be reached regarding cellular heterogeneity within chordoma, and to allow comparison of CAF populations with those found in other tumour types. Comparison of CAF functions in these predominantly mesenchymal tumours with epithelial solid tumours may reveal interesting lessons about the diverse phenotypes CAFs can bring to distinct tumour ecosystems. Understanding the role of CAFs in chordoma progression may also lead to therapeutic opportunities, but separation of correlation and causation in CAF regulation of tumour phenotypes remains a significant challenge. © 2025 The Author(s). *The Journal of Pathology* published by John Wiley & Sons Ltd on behalf of The Pathological Society of Great Britain and Ireland.

Chordomas are rare mesenchymal tumours that develop in the base of the skull, spine, or sacrum. They are widely understood to arise from remnants of the embryonic notochord, based on anatomic, histological, and molecular features. Despite their mesenchymal nature, single‐cell (sc) transcriptional analyses indicate retention of epithelial markers in malignant cell populations, expressed alongside mesenchymal and epithelial‐mesenchymal transition (EMT) markers [1, 2]. Cancer‐stem cell populations have also been described in multiple studies [1, 2]. Chordomas are typically characterised by brachyury (TBXT) dependence, indicative of their notochord origins, and high levels of TGFβ pathway activation. Chordomas contain numerous noncancerous stromal cells, dominated by subsets of heterogeneous cancer‐associated fibroblasts (CAFs) and diverse immune populations.

In a recent issue of this journal, Zheng and Guo [3] use scRNA sequencing to describe the presence of an abundant subset of inflammatory CAFs (iCAFs), which are associated with chordoma invasion and poor outcome (Figure 1). While the majority of CAF populations in solid tumours express a variety of extracellular matrix (ECM) and myofibroblast markers, iCAFs are distinguished by their abundant expression of inflammatory genes, including cytokines, chemokines, and inflammatory signalling components. Spatial transcriptomic and immunofluorescence studies indicate that these iCAFs are largely distal to malignant cells and associate with high levels of immune infiltrate, as has been described for iCAF populations in other solid tumours [4]. The authors propose that iCAFs drive tumour progression through various mechanisms, predominated by reciprocal paracrine interactions with immune cells. Using a powerful multiplexed quantitative immunofluorescence (QIF) dataset from 116 patients, with associated 10‐year outcome data, the authors show that high iCAF density associates with both clinicopathological features, including local invasion and adverse outcome; this is corroborated in bulk RNA‐seq datasets using an iCAF gene‐signature scoring system.

**Figure 1:**
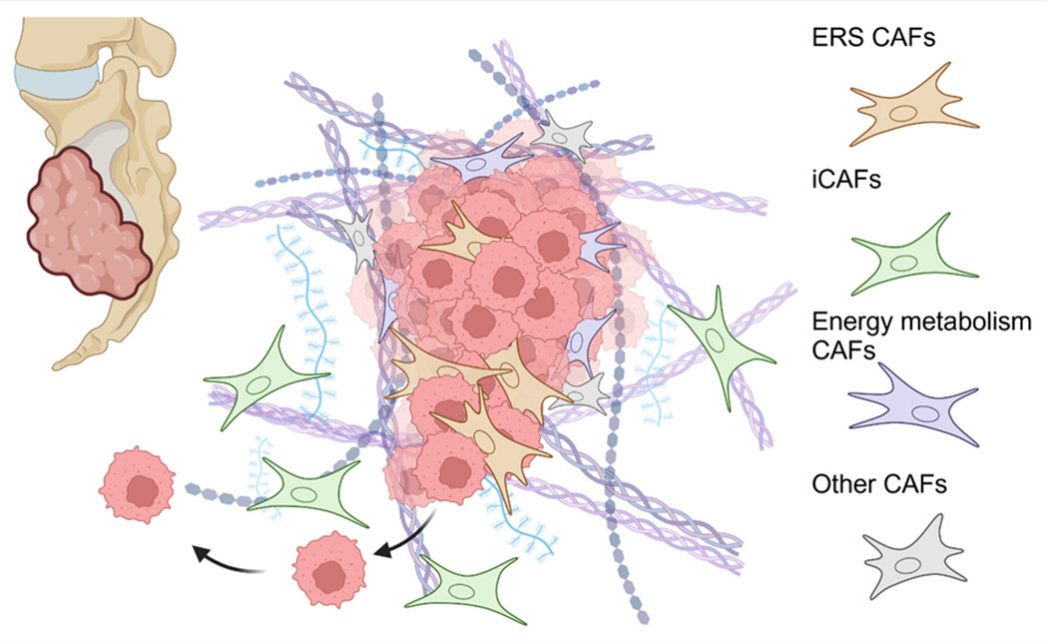
Distinct CAF subtypes arising in chordoma. Endoplasmic reticulum stress (ERS) and energy metabolism CAFs are localised in close association with chordoma tumour cells, in contrast to inflammatory iCAFs, which are not found in close proximity [2, 3]. In separate studies, both ERS CAFs and iCAFs are associated with tumour invasion and poor prognosis, albeit with distinct proposed mechanisms. Further, ERS CAFs are almost exclusively found in recurrent chordomas. Created in BioRender; Henry, J. (2025) https://BioRender.com/a94t585.

## Causation versus correlation for distinct CAF subtypes

Evidence for iCAFs populations, alongside myofibroblast CAFs and other subtypes, have been described in many solid tumour types, and association of inflammatory tumour signatures with poor outcome has been variously reported (reviewed in ref. [4]). However, there remain open questions about causal *versus* correlative relationships between distinct CAF subtypes and disease outcome. While iCAFs may be associated with sites of invasion, their phenotype could be driven by invasion and inflammation rather than playing a causative role. Further, co‐occurrence of multiple CAF types can make attribution of causation problematic. Indeed, a previous scRNA‐seq analysis of chordoma from the lead author of the current study helped define a distinct endoplasmic reticulum stress‐related CAF (ERS‐CAF) subpopulation, which also predicts invasion and poor outcome [2]. Critically, implication of this ERS CAF subtype in tumour progression, by examining transcriptomic signatures or through immunofluorescence, used the same tumour cohorts as the current study. In contrast to the iCAFs described here, ERS CAFs associate closely with malignant cells, where they are proposed to promote malignant tumour cell behaviour directly, through paracrine growth‐factor secretion, ECM modulation, and adhesion signalling (Figure 1). In stroma‐rich tumours, the abundance of distinct CAF subtypes is strongly correlated; myofibroblast CAF‐rich tumours tend to also be rich in iCAFs and immune cells. Which of these characteristics is critical for defining outcome is therefore difficult to untangle. Indeed, the current authors previously reported that chordoma outcome could be predicted by the tumour‐stroma ratio, without qualitative assessment of stromal content [5]. These parallel studies beg the question as to which CAFs are drivers and which passengers on the road to malignant progression.

Clues may be found in CAF origins and abundance across the disease time course. In the two separate CAF studies described above, chordoma CAF populations were compared with CAFs from nucleus pulposus samples, and little overlap was seen between the normal tissue resident fibroblasts and the chordoma‐associated CAFs. Pseudotime analysis suggests that all CAF subtypes in chordoma differentiate from resident fibroblasts, which progressively evolve into activated myofibroblast CAF populations, from which iCAFs eventually emerge. This concurs with powerful longitudinal studies in other cancers, including pancreatic cancer [6]. To complicate matters, myofibroblast ERS CAFs were almost exclusively found in recurrent chordomas, which might implicate prior therapy as a factor driving the ERS phenotype, in addition to endogenous signals. When it comes to attribution of adverse outcome to individual CAF subtypes, the picture remains unclear, as both ERS and iCAFs arise as tumours progress and both separately associate with invasion (Figure 1).

To address causation in the current study, *in vivo* data are presented where chordoma isolated iCAFs are shown to promote malignant behaviours *in vitro* and support tumour growth in a subcutaneous xenograft model; normal fibroblasts are used as a control in these studies. While encouraging, caution must be applied here. Co‐culture of malignant cells with CAF models has been widely shown to confer survival advantage and promote growth and invasive behaviour in many cancer models both *in vitro* and *in vivo*, but this has not always translated to appropriate genetic models. Perhaps lessons can be learned here from pancreatic ductal adenocarcinoma (PDAC), where targeting CAFs and the fibrotic microenvironment has been the subject of extensive work. Numerous studies have indicated that CAFs, derived from pancreatic stellate cells and resident fibroblasts, play diverse tumour supporting roles, including promotion of invasion and suppression of antitumour immunity. Surprisingly, however, ablation of α‐SMA expressing CAFs in spontaneously developing pancreatic tumours resulted in more aggressive metastatic disease [7]; this has been corroborated in other related pancreatic cancer models, with consensus suggesting that loss of myofibroblast CAFs can result in more inflammatory and aggressive tumours [4, 8]. Attributing causative functions to specific subsets of CAFs thus requires a more nuanced approach. As a useful example, Dominguez *et al* identified a minor subset of LRCC15+ CAFs in PDAC, which arise during late pancreatic tumour development [6]; specific targeting of this subset *in vivo* in a pancreatic tumour model (KPR; *Kras* ^*LSL.G12D/wt*^ *;p16/p19* ^*fl/wt*^ *;p53* ^*LSL.R270H/wt*^ *;Pdx1.Cre*) was effective at limiting tumour growth, while also promoting antitumour immunity and checkpoint inhibitor response [9]. Informatively, the aggressive inflammatory tumours observed with the deletion of α‐SMA‐positive CAFs also enhanced tumour response to anti‐CTLA4 checkpoint blockade [7]. Such studies highlight the need to separate correlation from causation, which will be critical when considering therapeutic targeting of the tumour stroma.

## Can targeting the stroma in chordoma provide clinical benefit?

To date, at least four separate studies have reported the single‐cell transcriptomic landscape of chordoma, alongside immune‐cell focussed studies (Table 1) [1, 2, 10, 11]. All studies concur on the identification of TBXT‐expressing cancer cells exhibiting high epithelial‐mesenchymal signatures and TGFβ pathway activation. Cancer‐stem cells and a highly complex immune infiltrate are also common features. Abundant fibroblast populations are identified in each of these datasets, although little effort has been made to cross‐compare the transcriptomic data. A more comprehensive description of the evolving CAF landscape during malignant progression, therapy response and disease relapse would provide important context for the development of stromal targeting strategies. As an example, the ERS CAFs described by Zhang et al are almost exclusively found in relapsed chordoma [2]. Relapse occurs in cases refractory to surgery and radiotherapy, where immunotherapy is emerging as a promising option. To what degree would ERS CAFs suppress antitumour immunity and would targeting these cells be useful? In contrast, iCAFs are shown to appear late in poor prognosis, primary invasive disease, and may play an important role in sculpting the immune infiltrate, with implications for immunotherapy responses. Combining data from multiple studies will be required to better understand commonalties and heterogeneity between CAF subtypes identified in chordoma.

**Table 1:** Cancer‐associated fibroblast subtypes identified in single cell RNA‐sequencing studies on chordoma.

CAF subtypes	Selected markers	Biological functions/notes	Samples/cancer type	References
4 CAF clusters: ‐ iCAFs (inflammatory) ‐ Energy metabolism enriched CAFs ‐ Cell junction enriched CAFs ‐ ECM CAFs	All clusters*: POSTN, THY1, ACTA2, FBLN1, FAP, PDGFRA* iCAF: *IL1B, HLA‐DRA, MMP9, CCL4*	iCAFs: tumour invasion, poor prognosis. Localised distant from cancer cells.	7 primary sacral chordomas 4 nucleus pulposus	Zheng 2025 [3]
4 CAF clusters: ‐ myCAFs (myofibroblast) ‐ ECM CAFs ‐ Ribosomal CAFs ‐ Fibroblast‐like	myCAF: ACTA2, *MYLK, MYL9* ECM CAF: *PDGFRA* *Fibroblast‐like: APOD and CYP1B1*	FN1, collagen and TGFβ interactions between CAFs and cancer cells; EMT/TGFβ pathway targeted.	6 primary skull base chordomas	Zhang 2022 [1]
6 CAF clusters: ‐ mCAFs – ECM/matrix ‐ iCAFs ‐ bone CAFs ‐ vascular CAFs (2 clusters) ‐ EMT CAFs	All clusters: ACTA2, Col1a2, PDGFRB mCAFs: collagens iCAFs: IL1RL1, CCL3, CCL4l2, CCL8	20% of cells are fibroblasts. TGFβ most prominent pathway driving invasion and immune suppression.	6 primary chordomas: ‐ 2 skull base ‐ 3 vertebrae ‐ 1 sacrum	Duan 2022 [10]
4 CAF clusters ‐ ERS CAFs ‐ Energy metabolism enriched CAFs ‐ 2 rare CAF clusters	All clusters: POSTN, ACTA2, FAP, PDGFRA ERS CAFs: ERS and Hypoxia signature genes, HSPs, VEGFA	Endoplasmic reticulum Stress (ERS) CAFs associated with recurrence, invasion, poor prognosis. ERS CAFs associate closely with cancer cells.	3 primary chordomas 3 recurrent chordomas 3 nucleus pulposus	Zhang 2024 [2]

Chordoma is a clinically important but relatively rare disease. It may therefore also be useful to cross‐compare CAF subsets with those arising in other mesenchymal bone tumours. No doubt the clusters identified in chordomas, and other related tumours, would share significant overlap, and this would allow advances in clinical trials in related tumour types to be more readily translated to chordoma. As in other cancers, the need to identify discriminatory biomarkers to define functionally distinct CAF types remains critical; the chordoma studies published here can support this endeavour. The rapidly advancing technology for examining spatial transcriptomics at single‐cell resolution also provides an increasing opportunity to explore the local relationships between these distinct stromal populations, the malignant components, and the immune landscape. Ultimately, this has the potential to define biomarkers to identify, and even target, cell types of therapeutic interest. A framework for integrating single‐cell and spatially resolved data from diverse studies will, however, be necessary to better define mechanisms of tumour progression and therapeutic vulnerabilities.

## Author contributions statement

AJMC and JCH conceived and wrote the article, compiled the table, and designed the figure.

## Data Availability

Data sharing is not applicable to this article, as no datasets were generated or analysed during the current study.

## References

[1] Zhang Q , Fei L , Han R , *et al*. Single‐cell transcriptome reveals cellular hierarchies and guides p‐EMT‐targeted trial in skull base chordoma. Cell Discov 2022; 8: 94. 36127333 10.1038/s41421-022-00459-2 PMC9489773

[2] Zhang TL , Xia C , Zheng BW , *et al*. Integrating single‐cell and spatial transcriptomics reveals endoplasmic reticulum stress‐related CAF subpopulations associated with chordoma progression. Neuro Oncol 2024; 26: 295–308. 37772937 10.1093/neuonc/noad173 PMC10836767

[3] Zheng BW , Guo W . Multi‐omics analysis unveils the role of inflammatory cancer‐associated fibroblasts in chordoma progression. J Pathol 2025; 265: 69–83. 39611243 10.1002/path.6369

[4] Menezes S , Okail MH , Jalil SMA , *et al*. Cancer‐associated fibroblasts in pancreatic cancer: new subtypes, new markers, new targets. J Pathol 2022; 257: 526–544. 35533046 10.1002/path.5926 PMC9327514

[5] Zou MX , Zheng BW , Liu FS , *et al*. The relationship between tumor‐stroma ratio, the immune microenvironment, and survival in patients with spinal chordoma. Neurosurgery 2019; 85: E1095–E1110. 31501892 10.1093/neuros/nyz333

[6] Dominguez CX , Müller S , Keerthivasan S , *et al*. Single‐cell RNA sequencing reveals stromal evolution into LRRC15^+^ myofibroblasts as a determinant of patient response to cancer immunotherapy. Cancer Discov 2020; 10: 232–253. 31699795 10.1158/2159-8290.CD-19-0644

[7] Özdemir BC , Pentcheva‐Hoang T , Carstens JL , *et al*. Depletion of carcinoma‐associated fibroblasts and fibrosis induces immunosuppression and accelerates pancreas cancer with reduced survival. Cancer Cell 2014; 25: 719–734. 24856586 10.1016/j.ccr.2014.04.005 PMC4180632

[8] Murray ER , Menezes S , Henry JC , *et al*. Disruption of pancreatic stellate cell myofibroblast phenotype promotes pancreatic tumor invasion. Cell Rep 2022; 38: 110227. 35081338 10.1016/j.celrep.2021.110227 PMC8810397

[9] Krishnamurty AT , Shyer JA , Thai M , *et al*. LRRC15^+^ myofibroblasts dictate the stromal setpoint to suppress tumour immunity. Nature 2022; 611: 148–154. 36171287 10.1038/s41586-022-05272-1 PMC9630141

[10] Duan W , Zhang B , Li X , *et al*. Single‐cell transcriptome profiling reveals intra‐tumoral heterogeneity in human chordomas. Cancer Immunol Immunother 2022; 71: 2185–2195. 35084549 10.1007/s00262-022-03152-1 PMC10992276

[11] Niu HQ , Zheng BY , Zou MX , *et al*. Complex immune microenvironment of chordoma: a road map for future treatment. J Immunother Cancer 2024; 12: e009313. 38908855 10.1136/jitc-2024-009313 PMC11328617

